# Supplementary material for: Knowledge, attitudes and practice towards yellow fever among nomadic populations: A cross-sectional study in yellow fever outbreak communities in Ghana
Source: PLOS Glob Public Health. 2023 Mar 16;3(3):e0000733. doi: 10.1371/journal.pgph.0000733 (PMC10019665; doi:10.1371/journal.pgph.0000733)
Supplement: S2 File — (DOCX) [file pgph.0000733.s002.docx]

**Household Questionnaire ON YELLOW FEVER:**

| **Details of the household** | | | |
| --- | --- | --- | --- |
| Date |  | Initials of fieldworker: |  |
| District: |  | Head of House (HoH) name: |  |
| Household No: |  |  |  |
| GPS Coordinates |  | Interviewee name, if not HoH: |  |

| **1. Socio-demographic characteristics of study participant (circle or tick as appropriate)** | | | |
| --- | --- | --- | --- |
| Q1. Age Group | <20 | Q2. Sex | Male |
|  | 20-24 |  | Female |
|  | 25-29 | Q3. Marital Status | Married |
|  | 30-34 |  | Single |
|  | 35-39 |  | Divorced |
|  | >40 |  | Widowed |
| Q4. Education | Illiterate/ None | Q5. Occupation | Unemployed / Housewife |
|  |  |  |  |
|  | Primary |  | Farmer |
|  | Secondary |  | Trader/Vendor |
| Q6. How many people living in your household? (including participant) | 4 |  | Herdsmen |
|  | 5 |  | Agro pastoralist |
|  | 6 |  | Student |
|  | Other: |  | Other: |
| Q7. Religious affiliation | Islam  Christian  Traditional | Q8. Nationality? | Ghanaian  Foreigner |
| Q9. If foreigner, which country? |  | Q10. **Health insurance status** |  |

| **2.** **Knowledge of yellow fever (YF) symptoms, signs and transmission modes (circle as appropriate)** | | | | | |
| --- | --- | --- | --- | --- | --- |
| Q7. Have you heard of yellow fever (YF)? (**show the Card**)(Screening question) | Yes | | No | | |
| **2.1 Signs and symptoms** | | | | | |
| Q8. Is fever a symptom of YF? | Yes | No | | | Don’t know |
| Q9. Is headache a symptom of YF? | Yes | No | | | Don’t know |
| Q10. Is jaundice a symptom of yellow fever? | Yes | No | | | Don’t know |
| Q11. Is muscle pain a symptom of YF? | Yes | No | | | Don’t know |
| Q12. Is rash a symptom of YF? | Yes | No | | | Don’t know |
| Q13. Is bloody vomiting a symptom of YF? | Yes | No | | | Don’t know |
| Q14. Are there any other symptoms that have not been listed? (Please specify) |  | | | | |
| **2.2 Transmission** | | | | | |
| Q15. Do mosquitoes transmit YF?  IF No, what transmit YF? | Yes | No | | | Don’t know |
| Q16. Are they the same mosquitoes that transmit malaria? | Yes | No | | | Don’t know |
| Q17. Does ordinary person to person contact transmit YF? | Yes | No | | | Don’t know |
| Q18. Is YF transmitted through food and water? | Yes | No | | | Don’t know |
| Q19. When are the YF vector most likely to feed/bite? | Night | Day | | Both | Don’t Know |
| Q20. Do the YF mosquitoes breed in standing water? | Yes | No | | | Don’t know |
| Q21. Can mosquitoes breed inside the home? | Yes | No | | | Don’t know |
| Q22. Does removal or covering of standing water prevent mosquito breeding? | Yes | No | | | Don’t know |
| Q23. Can pouring chemicals into standing water kill mosquito larvae? | Yes | No | | | Don’t know |

| **3.** **Attitudes towards yellow fever** | | |
| --- | --- | --- |
| Q24. Is yellow fever a serious illness? | Yes | No |
| Q24b. If yes, why: | | |
| Q25. Are you at risk of YF where you live? | Yes | No |
| Q25b. If yes, why: | | |
| Q26. What disease are you most fearful of getting? | Malaria | Typhoid |
|  | Yellow Fever | Bacterial infection |
|  | Pneumonia | Regular fever |
|  | Other (please specify): | |
| Q27. Is controlling the breeding sites of mosquitoes a good strategy to prevent YF? | Yes | No |
| Q28. Is vaccination a good strategy to prevent YF? | Yes | No |
| Q29. Do you think communities should actively participate in controlling the mosquitoes of YF? | Yes | No |
| Q30. Do you think it’s the responsibility of the administrative & health office to control and prevent YF? | Yes | No |

| **4. Preventative practices against yellow fever** | | | | | |
| --- | --- | --- | --- | --- | --- |
| **4.1 Preventing mosquito-man contact** | | | | | |
| Q31. Do you do anything to reduce mosquitoes? (If yes, please answer the questions below. If no, then proceed to “*Eliminating breeding sites”*) | | Yes | | No | |
| Q31a. Has the government come to spray insecticide to reduce mosquitoes? | | Yes | | No | |
| Q31b. Do you prevent standing water around the house to reduce mosquitoes? | | Yes | | No | |
| Q31c. Do you use insecticide treated nets to protect against mosquitoes in the home? | | Yes | | No | |
| Q31d. Do you use smoke to drive mosquitoes away? | | Yes | | No | |
| Q31e. Do you cover your body with clothes to protect against mosquitoes? | | Yes | | No | |
| **4.2 Eliminating Breeding Sites** | | | | | |
| Q32. Do you cover water containers in the home? | Yes | | | No | |
| Q33. How often do you clean water filed containers and ditches around the house? | Everyday | | Once a week | Once a month | Never |
| Q34. Do you turn containers upside down to avoid water collection? | Yes | | | No | |
| Q35. Do you do anything else to protect against mosquitoes? (please specify) | | | | | |

| **(5) Sources of information regarding yellow fever** | | | | |
| --- | --- | --- | --- | --- |
| Q36. Where do you normally get your information about yellow fever? | TV | Radio | Health Extension Workers | Religious Leader |
|  | Loudspeaker | Brochure | Friends | Internet |
|  | Other: | | | |

| **(6) Yellow Fever Case Finding** | | |
| --- | --- | --- |
| Q37. Have you ever had malaria? | Yes | No |
| Q38. Have you ever had YF? | Yes | No |
| Q38b. If yes, when: | | |
| Q38c. If yes, what treatment did you receive and from whom (health centre / hospital / traditional healer / other) | | |
| Q39. Do you know anyone who has had YF? | Yes | No |
| Q39b. If yes, who and when: | | |
| Q39c. If yes, what is their profession? | | |

| **(7) Vaccination Coverage Estimation** | | |
| --- | --- | --- |
| Q40. Have you ever been vaccinated against YF? | Yes | No |
| Q40b. If yes, do you remember where or when you had it? (Was it at your health post?) | | |
| Q41. Has anyone else in your household been vaccinated against YF? | Yes | No |
| Q41b. If yes, who and when? (Please name ALL members of your household who have been vaccinated, their ages and any other details e.g. where, when) | | |

| **(8) Other epidemiological risk factors** | | |
| --- | --- | --- |
| Q42. Do you work in the forest areas? | Yes | No |
| Q43. Have you had any contact with monkeys? | Yes | No |
| Q44. Is there presence of false banana around the home? | Yes | No |
| Q45. Do you store water in open-containers inside or outside the home? | Yes | No |
| Q46. Have you (and your family) recently migrated to this area? | Yes | No |
| Q46b. If yes, from where, when and why? | | |
| VACCINATION STATUS | | |
| Q1.How many people live in your household including yourself? |  |  |
| Q2.How many how many household members are 9 years and older? |  |  |
| Q3.How many of them have received yellow fever vaccination? |  |  |
| Q4.Number of household members with the yellow fever vaccination card |  |  |
| Q5.Number of household members with verbal reporting of yellow fever vaccination? |  |  |
| Q6.How many household member have not received yellow fever vaccination? |  |  |
| Q7. Reasons for mot receiving the vaccine? | Having travelled out of district  Lack of transport to vaccination posts  Being sick during the vaccination campaigns  Being at school during the campaigns  Long waiting time at vaccination posts  Rudeness of health workers at vaccination posts  Under aged for the vaccines | |
| Q8. Where did you receive the yellow fever vaccination? | In this community  In another community  In another country  As part of the immigration process | |
| Q9. If part of the immigration process. Which country are you coming from? |  |  |
| Q10. How long have you been in this country? |  |  |
| Q11.How many household members have valid health insurance? |  |  |
|  | | |

Do you have any other questions or comments you would like to make?

____________________________________________________________________________________________________________________________________________________________________________________________________________________________________________________________________________________________________
