# Supplementary material for: Knowledge, attitudes and practice towards yellow fever among nomadic populations: A cross-sectional study in yellow fever outbreak communities in Ghana
Source: PLOS Glob Public Health. 2023 Mar 16;3(3):e0000733. doi: 10.1371/journal.pgph.0000733 (PMC10019665; doi:10.1371/journal.pgph.0000733)
Supplement: Attachment — Submitted filename: response to reviewer comments v2.docx [file pgph.0000733.s003.docx]

**Author response to reviewer comments**

We thank all reviewers and the editor for their very helpful comments. Please see below tables that address each comment individually.

**Journal Requirements/Editor feedback**

| 1. Please provide your detailed Financial Disclosure statement. This is published with the article. It must therefore be completed in full sentences and contain the exact wording you wish to be published.  a. Please clarify all sources of funding (financial or material support) for your study. List the grants (with grant number) or organizations (with url) that supported your study, including funding received from your institution.  b. State the initials, alongside each funding source, of each author to receive each grant. c. State what role the funders took in the study. If the funders had no role in your study, please state: “The funders had no role in study design, data collection and analysis, decision to publish, or preparation of the manuscript.” d. If any authors received a salary from any of your funders, please state which authors and which funders.  If you did not receive any funding for this study, please simply state: “The authors received no specific funding for this work. | Financial disclosure statement has been added into the manuscript |
| --- | --- |
| 2. Please provide separate figure files in .tif or .eps format only and remove any figures embedded in your manuscript file. Please also ensure that all files are under our size limit of 10MB. | Figure 1 is now provided as a .tif file |
| 3. Figure 1: please (a) provide a direct link to the base layer of the map (i.e., the country or region border shape) and ensure this is also included in the figure legend; and (b) provide a link to the terms of use / license information for the base layer image or shapefile. We cannot publish proprietary or copyrighted maps (e.g. Google Maps, Mapquest) and the terms of use for your map base layer must be compatible with our CC-BY 4.0 license.  Note: if you created the map in a software program like R or ArcGIS, please locate and indicate the source of the basemap shapefile onto which data has been plotted.  If your map was obtained from a copyrighted source please amend the figure so that the base map used is from an openly available source. Alternatively, please provide explicit written permission from the copyright holder granting you the right to publish the material under our CC-BY 4.0 license.  Please note that the following CC BY licenses are compatible with PLOS license: CC BY 4.0, CC BY 2.0 and CC BY 3.0, meanwhile such licenses as CC BY-ND 3.0 and others are not compatible due to additional restrictions.  If you are unsure whether you can use a map or not, please do reach out and we will be able to help you. The following websites are good examples of where you can source open access or public domain maps:  * U.S. Geological Survey (USGS) - All maps are in the public domain. (<http://www.usgs.gov>)  * PlaniGlobe - All maps are published under a Creative Commons license so please cite “PlaniGlobe, <http://www.planiglobe.com>, CC BY 2.0” in the image credit after the caption. (<http://www.planiglobe.com/?lang=enl>)  * Natural Earth - All maps are public domain. (<http://www.naturalearthdata.com/about/terms-of-use/>) | The map was drawn within our research group.  We have added text to the figure legend (as per other PLOS submissions) –  “*Image drawn within our research group, the Clinical Informatics Research Unit at University of Southampton”* |
| Reviewer 3 has raised concerns about the rigor of the statistical analysis and reporting as well as the use of standard English. Please pay special attention to these comments in your revisions. Additionally, the authors should review the appropriate reporting guidelines (<https://journals.plos.org/globalpublichealth/s/submission-guidelines#loc-guidelines-for-specific-study-types>) and criteria for publication (<https://journals.plos.org/globalpublichealth/s/criteria-for-publication>) to ensure they have included all the appropriate information about they study before resubmission. | We have made several revisions to the manuscript, reflected in the comments below.  We have also made several revisions to the standard of English throughout the manuscript. |

**Reviewer 1**

| Title: Remove the first Population since there is another term population in the title. New title: Knowledge, attitudes, and practice towards Yellow Fever among nomadic populations: A cross-sectional study in Yellow Fever outbreak Communities in Ghana. | Thank you, we have removed the first ‘population’, as suggested here |
| --- | --- |
| Figure 1": The quality is bad. I suggest for the authors to submit a high resolution so that the labels are readable. Please include a map scale. Indicate the sampling sites or households as points or dots. It is also best to provide administrative boundaries if needed. | We disagree the figure is ‘bad’. The file image is already high resolution. It is included as the readerships of PLOS Global Health is international from all corners of the world, so highlighting the location of the study site in this way is useful for some readers.  However, we do agree that a further map more specific to the West Gonja area is a useful idea. The PLOS rules on maps are tight, and thus we are unsure about what map of West Gonja we can safely use that does not breach copyright etc. Thus, we seek editorial guidance on this.  A granular map of the households is a nice idea, but does not exist. |
| Page 11: Data Analsis: The data collected "from the _____" was exported to Microsoft Excel 2019 for cleaning. | We have corrected this sentence. It now reads “Data was exported into Microsoft Excel 2019 for cleaning” |
| Page 11: Data Analysis: For the scores of each dependent variables, I suggest to create a table. | We agree. We have created a table for the outcome variables |
| Page 11 and 12: On the other hand, the key independent variables are __________. | We have a sub-heading of ‘independent variables’, followed by the text. So, to avoid any difference in writing style, we have made no changes here. |

**Reviewer 2**

| 1) Under the discussion, it would be good if the authors can compare and contrast their findings on Knowledge, attitude and practice (KAP) between yellow fever and other diseases such as dengue fever based on the following study:  Knowledge, Attitude and Practice about Dengue Fever among Patients Experiencing the 2017 Outbreak in Vietnam. Int J Environ Res Public Health. 2019 Mar 18;16(6):976. doi: 10.3390/ijerph16060976. PMID: 30889912; PMCID: PMC6466316. | We agree that this is a useful citation, and have included it in the discussion with brief commentary. |
| --- | --- |
| 2) Participants from Ghana from stay in rural areas and from lower social class. Please discuss strategies from other infectious studies (e.g. COVID pandemic) to improve KAP based on the methods proposed by following studies and include in the discussion:  Strengthen health care system in resource scarce setting:  Strengthening Health System and Community Responses to Confront COVID-19 Pandemic in Resource-Scare Settings. Front Public Health. 2022 Jul 6;10:935490. doi: 10.3389/fpubh.2022.935490. PMID: 35875028; PMCID: PMC9296814.  Involvement grassroot system: Feasibility of Intersectoral Collaboration in Epidemic Preparedness and Response at Grassroots Levels in the Threat of COVID-19 Pandemic in Vietnam. Front Public Health. 2020 Nov 17;8:589437. doi: 10.3389/fpubh.2020.589437. PMID: 33313040; PMCID: PMC7707108.  Involvement of village health collaborators:  Reaching further by Village Health Collaborators: The informal health taskforce of Vietnam for COVID-19 responses. J Glob Health. 2020;10(1):010354. doi:10.7189/jogh.10.010354 | We agree that brief mention of KAP around COVID-19 would be useful.  And thank you for the suggested references. However, we have integrated Ghana-specific citations into the discussion, highlighting the need for understanding of public behaviour and attitudes around covid-19 and routine health service delivery such as mass drug administrations. |

**Reviewer 3**

| 1. The quality of writing should be improved. The manuscript needs extensive stylistic and English editing to correct several grammatical and typographical errors. For instance, capitalized letters in 1st line in abstract: Global; 4th paragraph of abstract: Age, Nationality; duration of in (stay is missing); 5th paragraph of abstract: Nationality, Positive; Introduction: jaundice body pain, all subsections titles and several variables in all tables, etc. Some other mistakes are indicated in minor revision. | Thank you, we have made these (and other) changes, and also reviewed and made corrections as per other comments from this reviewer (see below) |
| --- | --- |
| 2. Statistical analysis and results reporting and presentation should also be improved. For instance:  2.1. Reporting of results in abstract should be improved. Add statistics for significant factors (predictors) associated with positive attitude and positive practices, where applicable. 2.2. Why predictors of poor or good knowledge were not identified? 2.3. Table 4: Why all variables were included in the multivariate analysis? This should be explained. 2.4. Table 5: Why all variables were included in the multivariate analysis? This should be explained. | Thank you, we do agree with you. We have inserted statistics to significant factors.  Regarding the use of all variables in the multi-variare analysis - This is because, some insignificant variables in bivariate regression model may be significant in multivariate regression model. This is termed as suppression effect. The classical example is shown in table 4. Age group was not significant in bivariate analysis but significant in Multivariate regression. |
| 3. Discussion section is too lengthy and should be entirely rewritten. It should be shortened substantially focusing on important findings and the important message to policy-makers. Over repetition of results should be avoided. | Thank you, we do agree.  To summarise here, we have deleted chunks of text that are repeating earlier points. There is significant rewriting to improve the readability, hopefully making the discussion more concise. See the ‘tracked changes’ version of the manuscript for the edits made. |
| ABSTRACT: 5. Results in the 4th paragraph and 5th paragraph: it should be indicated if these results were obtained by univariate or multivariate analyses. Significant associations or not? Add statistics. | Thank you. All significant variables were obtained from a multivariate analysis  We have inserted  “Significant statistics were obtained from multivariate analysis” |
| 6. 5th paragraph: what was the most commonly used strategy? | We have corrected the sentence. It now reads,  “with 97.3% controlling mosquitoes in their households” |
| 7. Last paragraph: “….. yellow fever epidemic control can be improved in hard-to-reach communities through locally-tailored education and health promotion campaigns …” The conclusion is not supported by the findings. Yes, the population had poor knowledge about yellow fever, and this needs proper intervention; however, the impact of any intervention like health education is still not evaluated. | Yes, we do agree. New conclusion reads as  “Our findings show that yellow fever KAP was variable with clear knowledge gaps. Regular locally-tailored education and health promotion campaigns should be considered to improve knowledge and preventive practices against this infectious disease.” |
| INTRODUCTION: 8. 3rd sentence: rephrase to improve clarity. | We have simplified that sentence to  “There are an estimated 29,000-60,000 deaths globally each year.” |
| 9. “The virus”: which virus? | Revised to ‘the yellow fever virus’ |
| 10. 2nd paragraph: “in the continent”. Which continent? | Rephrased to ‘the African continent’ |
| 11. 6th paragraph: “In Iran, ….”. This paragraph should be rewritten to provide better justification of the study. | We have rephrased it to  “A multi-country comparison found African participants to typically have more knowledge of yellow fever compared to those from the East Mediterranean and other countries, with males and older age groups having highest knowledge” |
| 12. Information on the control program in Ghana should be provided? | We have added this text into the introduction  “Yellow fever control in Ghana is based upon tested of suspected cases, field investigations to determine likely vaccination status of exposed individuals, with vaccination campaigns sometimes deemed necessary to bring any outbreak under control” |
| METHODS: 13. Study Area, Design and Period: Areas’ epidemiological features related to vector-borne infections can be added. | We have added, with a citation, this text  “The Savannah region is humid, with the forest and accompanying water sources providing a breeding ground for mosquitoes. Transmission of malaria also high in the area throughout the year” |
| 14. Study Population, Participants, and inclusion Criteria: 2nd paragraph “Using the yellow fever line list ……. were purposively selected for the study.” This should be moved to previous section (study area). Information on invitations to participate, acceptance or rejection and related numbers should be added. | All household heads invited to participate, accepted our invitation.  We have added,  “All household heads invited to participate in the study accepted our invitation” |
| 15. “Using snowballing approaches,”: does this mean that other population groups resided in the targeted areas? | Yes, there are other resident populations, hence the approaches used to identify further potential participants who met the inclusion criteria.  We have slightly adjusted the sentence to read  “Using snowballing approaches, consented household heads/spouses were also asked to identify other nomadic households who may meet the inclusion criteria. “ |
| 16. “A total population of 403 nomadic households”: please rephrase. | Sentence is rephrased to  “Required sample size was 403, at a 95% confidence level.” |
| 17. Key dependent variables were; knowledge, attitudes and practices towards yellow fever. This should be merged with related paragraph. | This has been done |
| 18. “were considered as having “Positive knowledge”,” can be changed to “Good knowledge”. | We have made this change |
| 19. This scoring system is adapted from previous similar studies. (13). But only a single study was cited. | We have revised it to  “This scoring system is adapted from a previous similar study” |
| 20. 24 variables; 12 variables, using 9 variables. These can be items or questions. | We agree with you. We replaced variables with items.  We have also added  “(two items have more than 1 correct answer)”  Under knowledge, 24 items were used obtain knowledge score  These 2 variables below contained more than 1 right answer. Each correct answer was scored 1 or 0  “Signs and Symptoms of YF include” “Breeding Site for yellow fever vector includes” |
| 21. 60% cut-off point: How the 60% was calculated? The scores were either 1 or 0. Explain. | A score of 1 was given to correct response, 0 incorrect response. Maximum score for the 12 attitude items will be 12.  To get 60% cut of point (0.6*12)=7.2  All those who score 7.2 or more were considered Positive Attitude and less than 7.2 were considered Negative attitude |
| RESULTS: 22. Table 1: define HH Size in footnote. Religious affiliation can be removed and indicated in the text. | We have clarified the mention and definition of HH (household size) in the table.  And removed the religion row, and added mention into the text as suggested. |
| 23. In this study, only those who have heard of yellow fever were included the study. This is a vague statement. Does it mean that all the 414 participants heard about yellow fever? Did you ask at the beginning and only recruit those who had prior knowledge? This should be clearly explained. | All participants were screened with the question; **“I have heard of yellow fever before?”** at the start of the interview  Only those who responded yes were included in this study.  Those who have never heard of yellow fever were not included and the information were not captured by the authors |
| 24. “… a bite of an infected mosquito” or a bite of mosquito? | As per table 2, the reference is to “bite of an infected mosquito” |
| 25. About 47% believed yellow can be … Another example of writing mistakes. | We have corrected this to  “47% believed yellow fever can be…” |
| 26. Table 2: Monkey transmit YF to person. Revise! | We have revised this to  “can a monkey transmit YF to a person” |
| 27. YF can breed inside homes. Revise! | Revised to  “Can the YF vector breed inside homes” |
| 28. Overall knowledge was stated in a mean score. Why this was not following attitude and practice method? | Categorization of Knowledge, Attitude and practice were done in relation to similar studies. These studies have been cited. |
| 29. Nomadic Attitudes towards Yellow Fever: Some of the questions are related to knowledge and not to attitude. | We agree with you.  Some of the questions were lengthy so we tried shorten them to be presentable.  We have revised them. |
| 30. Table 3 (last variable) shows that 80.2% had negative attitude. This contradicts the results in previous text and in abstract “Over 80% of household heads surveyed had positive attitudes”. | Thank you. We have made a correction to Table 3.  From the analysis, 80.2% have positive Attitudes and Negative attitudes is 19.2% |
| 31. Overall Attitudes toward yellow fever: Table 4. But it is displayed in Table 3. Does this analysis include all participants or only those with | Overall Attitudes is in Table 3 and not Table 4  We have corrected it |
| 32. Table 4: (n) of each category must be added. | Thank you for the suggestion.  Logistics regression output can still be presented without (n) for each category added, as pre previous papers in PLOS Global Public Health (E.g. <https://doi.org/10.1371/journal.pgph.0001181>)  If editorial input is to revise the table further, we are happy to do so. |
| 34. Why males had lower level of correct or positive practices compared to females, although they have significantly higher level of positive attitude? | Yes, this is possible. It means their positive attitudes does not translate into practice. This explains why all participants had poor knowledge. Lower positive practices were influence by poor knowledge |
| DISCUSSION: 35. Comparisons with previous findings should be justified and explained. For example, reference #13 cited in the 3rd line can be invalid as the study was among university students and in Ethiopia. | We have revised the sentence to read as follows  “Our findings are similar to previous research in different populations, (13) where an Ethiopian study found in which only 9.6% of the university student participants were found to have adequate overall knowledge of yellow fever.”  There are several other revisions across the discussion, including clarification about the populations involved in the research that we cite |
| 36. “This affirms the fact that yellow fever is endemic in Ghana (3).” This and subsequent related sentences can be removed. | Agreed, this paragraph has been significantly revised, along with preceding and subsequent paragraph. So there may be a little context lost by only citing this ‘new paragraph’ here, but this is how the new section reads  “When considering attitudes, we found high (65.5%) awareness of the recent yellow fever outbreak among the nomadic population. Reported awareness has been high in Ethiopian studies, reporting 83% and 86% awareness, but only 25% awareness from an Indian study, It will therefore be good for Ghanaian health stakeholders to take advantage of the high awareness among the nomadic population to scale up interventions to help eradicate or reduce the incidence of YF.” |
| 37. Conclusion: It is important to ensure that there is good population knowledge, attitude and practice …. Can be removed. What are the important findings? What are the implications? What are your recommendations? | **We have revised that part of the conclusion to “**It is important to ensure that there is sustained health promotion among communities where there may be low existing vaccine uptake, raising awareness of yellow fever and the importance of immunization.” |
| 38. Figure 1: The current figure is not useful. The study areas should be indicated on the map. Regions’ boundaries should be displayed. | As per other reviewer comment, we are keen to keep this current map image. Much of the PLOS Global Health readership will be from outside Ghana and thus highlighting the study location will be useful information.  However, we do agree that a more specific map will be a useful addition.  The PLOS rules on maps are tight, and thus we are unsure about what map of West Gonja we can safely use. Thus, we seek editorial guidance on this. |
| 39. Reference list should be prepared following the journal’s instructions and style. | We have made changes to the content and formatting of the references. If editorial input is that there are further changes needed, then we’d be happy to make them. |
